# An assessment of the nutritional status of internally displaced school children in the West and Littoral Regions of Cameroon

**DOI:** 10.1002/fsn3.4068

**Published:** 2024-04-21

**Authors:** Aba Richard Ejoh, Boh Mirabelle Nwachan, Ngangmou Theirry Noumo

**Affiliations:** ^1^ Nutrition, Food and Bioresource Technology University of Bamenda Bamenda Cameroon

**Keywords:** Cameroon, internally displaced schoolchildren, nutritional status, West and Littoral Regions

## Abstract

Malnutrition remains a major public health challenge among children in developing countries, especially those experiencing civil wars and political unrest. It is imperative to ascertain the level of malnutrition, particularly in displaced children, to develop and effectively implement interventions. This study was a cross‐sectional survey conducted to assess the nutritional status of 657 internally displaced school‐aged children (5–15 years) enrolled in primary schools in the West and Littoral Regions of Cameroon. The height, weight, and mid‐upper arm circumference of the children were measured using standard measurements. Clinical examinations were also made on the children. Other parameters that affect nutritional status, such as morbidity and health‐seeking practices, were assessed using pre‐tested, structured interviewer questionnaires. An analysis of the data revealed that stunting was the most prevalent form of malnutrition (27.1%), followed by wasting (23%), thinness (21.6%), and underweight (20.1%). About 44.5% of the children had low serum iron, and 35.7% of them had poor protein status. The prevalence of anemia was 30.0%. The most common signs of protein, vitamin A, and iron deficiencies observed were thin, dry, or sparse hair (5.2%), pallor (7.3%), and xerosis (3.3%), respectively. About 32% of the children had been sick within the previous month of the study, and the most common illness was the common cold (19.8%). Most of them (>60%) had good health‐seeking practices. The high prevalence of stunting, wasting, thinness, underweight, anemia, protein, and iron deficiency calls for interventions such as nutrition education and supplementation to prevent malnutrition and diet‐related diseases among children.

## Graphical abstract

A cross‐sectional survey was conducted to assess the nutritional status of internally displaced school‐aged children in primary schools in the West and Littoral Regions of Cameroon. The results show a high prevalence of stunting, wasting, thinness, underweight, anemia, protein, and iron deficiency.

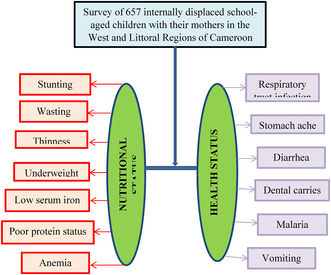

## 1 BACKGROUND

Malnutrition is a main public health problem in less developed countries, particularly those suffering from social insecurity, civil conflicts, and political instability. It has been linked to morbidity and mortality, as it is related to five of the 10 principal causes of death in less developed countries (FSIN, 2021). It has measurable adverse consequences for tissues, functions of the body, physical appearance, clinical performance, and obstetric functions. Children are the most obvious victims of malnutrition. Malnutrition during childhood is also associated with decreased resistance to infection, impairment of intellectual development and learning capacity, functional deterioration in adulthood, decreased work competence, and reduced financial output of the individual (Khan et al., 2017; WHO, 2017). Malnutrition, “the silent emergency,” is a conspirator, with at least 5.45 million child deaths recorded every year (UNICEF/WHO/World Bank Group, 2018).

Undernutrition is the most prevalent form of malnutrition, especially in developing countries (UNICEF/WHO/World Bank Group, 2018). It may be expressed in the form of protein‐energy malnutrition (PEM), which includes wasting, underweight, stunting, and/or micronutrient malnutrition. PEM is the most fatal type of malnutrition globally. About one‐quarter of all children worldwide are affected by PEM (UNICEF/WHO/World Bank Group, 2018).

An evaluation of trends in malnutrition in the countries of Africa reveals that the rates of undernutrition are increasing instead of dropping to attain the global target for 2025, which is to decrease the percentage of children who are stunted by 40% (WHO, 2017). Undernutrition is highly prevalent among children in Cameroon, as the rate of stunting among children <5 years old is 31%, underweight is 16%, and 5.2% of them are wasted (Global Nutrition Report, 2018). The prevailing nutritional status of children in the two regions from which the study children were displaced (the Northwest and Southwest Regions) is worrisome, as 4.4% and 5.6% of children in the Northwest and Southwest Regions, respectively, are suffering from global acute malnutrition (GAM) (OCHA, 2022). This prevailing trend of malnutrition is nowhere near that of the Sustainable Development Goals, whose goal is to stop all types of malnutrition by 2030 by terminating hunger, attaining food security and upgraded nutrition, and fostering sustainable agriculture (United Nations, 2016).

The school‐age period is nutritionally very significant, and deficiencies are of major concern because school age is a dynamic growth stage, as well as mental development (Wrottesley et al., 2022). Studies have shown that health complications due to poor nutritional status in school‐age children are among the greatest widespread causes of low school turnout, nonattendance, early dropout, and poor classroom outcomes (Teo et al., 2021). In school‐aged children, thinness can result in delayed maturity, deficits in muscle potency and work competence, and decreased bone solidity later in life. Iron deficiency in school‐age children is associated with delay in growth, increased vulnerability to diseases, and poor mental development, resulting in a lower intelligence quotient and behavioral anomalies that reduce the quality of life of the individuals concerned (Zerga et al., 2022).

Conflict unavoidably results in damage to lives, substantial injuries, extensive psychological anguish, an exacerbation of existing malnutrition among vulnerable persons (especially children), and outbursts of infectious diseases, particularly among persons who are internally displaced (Loewenberg, 2015). Forcible displacement of populations usually affects health systems as it lessens people's political security and restricts their food accessibility, medications, portable water, hygiene, shelter, and access to health care services, which may result in malnutrition. Internally displaced persons are among the most vulnerable of forced migrants and often suffer intensely from several types of malnutrition (Dago, 2021).

Populations affected by an emergency often live in difficult conditions. This makes them vulnerable to malnutrition. IDPs often arrive in camps in a nutrient‐depleted state, and most of them are fully dependent on emergency food rations (Dago, 2021). Such populations often have little access to local markets, and even if they do, they often have little purchasing power to buy micronutrient‐rich foods to supplement their diet. Also, in emergencies, the general deterioration in nutritional status, loss of access to traditional foods, and lack of dietary diversity further exacerbate micronutrient deficiencies in the affected population (Dago, 2021). Additionally, growing vegetables and fruit, which are micronutrient‐rich foods, is often limited by land and water availability, hence limiting access to fresh foods (Prinzo & de Benoist, 2002). While forcible displacement is the foremost cause of malnutrition, its consequences, such as inadequate supplies of food, and communicable diseases, may also contribute to the deterioration of the condition. Moreover, previous studies revealed that IDP children are more likely to suffer from diseases such as malaria, diarrhea, and measles and, consequently, mortality (Vos et al., 2020).

Nutritional status is affected by dietary intake and the occurrence of infections (Kathryn & Begum, 2011). The examination of anthropometry is a compulsory instrument to evaluate the health and nutritional status of children, who are the cornerstone of a nation (WHO, 2017). It is imperative to provide high‐quality data on the nutritional and health status of children, particularly those who are forcibly displaced, as it would aid in prioritizing and formulating precautious nutrition intervention programs that are evidence‐based, ensuring that the nutritional problems that are of substantial concern are targeted (Development Initiatives, 2017).

Numerous studies have shown that children who are affected by conflicts face a disproportionate problem of malnutrition and poor health (Akeh et al., 2022; Bougma et al., 2022; Chidiogo et al., 2022; Salami et al., 2020; Vos et al., 2020). Since 2016, the Northwest and Southwest Regions of Cameroon have been experiencing armed conflicts associated with insecurity and the operation “no school”, which led to the displacement of approximately 679,393 people by the end of 2022, and about 43% of the displaced persons are children (OCHA, 2022). Most of the IDPs are located in the Littoral, Central, and West Regions of Cameroon (OCHA, 2022). Some of the displaced people live in rented houses, while others are staying in overcrowded houses with host families, and others live in “critical shelter” without mosquito nets, resulting in a general increase in malaria and flu syndrome. Food security, shelter, and education were expressed as the top three priority needs of the displaced pupils (OCHA, 2019). To understand the full extent of this problem of displacement among schoolchildren in Cameroon, a reliable assessment of nutritional status is required. Yet, to the best of our knowledge, data on the nutritional status of internally displaced schoolchildren in Cameroon has not been published.

The purpose of this study was therefore to determine the nutritional and health status of internally displaced schoolchildren in the West and Littoral Regions of Cameroon through a participatory baseline survey.

## 2 MATERIALS AND METHODS

### 2.1 Study area

The West Region is located in the central‐western part of the Republic of Cameroon, and its headquarter is Bafoussam. It is the smallest of the ten regions of Cameroon, with a surface area of 14,000 km^2^. However, it has the greatest population density. It is divided into eight divisions. The West Region shares a boundary with the Northwest Region to the northwest, the Adamawa Region to the northeast, the Centre Region to the southeast, the Littoral Region to the southwest, and the Southwest Region to the west (Benneh & DeLancey, 2004).

The Littoral Region is located in the southwest of the country, with its capital in Douala. It has a population of 3,354,978 people and a surface area of 20,248 K. It is bordered to the north by the West Region, to the east by the Central Region, to the south by the South Region, and to the west by the South West Region. The Littoral Region is divided into four divisions namely: Moungo, Nkam, Sanaga‐Maritime, and the Wouri Divisions (Benneh & DeLancey, 2004). The Moungo and Wouri Divisions were purposely chosen for the study because these divisions host the greatest number of IDPs (45,000 and 31,880, respectively) compared to the other divisions (OCHA, 2022).

### 2.2 Study design

A cross‐sectional study was carried out in the West and Littoral Regions of Cameroon. A total of 10 and 12 schools, respectively, were randomly subsampled from the West and Littoral Regions for the study. The sample size was calculated using Fischer's formula for sample size calculation (Fisher et al., 1991), considering the prevalence of underweight among children between the ages of 5 and 19 years in Cameroon is 24% (Global Nutrition Report, 2018). The degree of accuracy desired was set at 5% or a 95% confidence level. A non‐response rate of 10% was considered. The calculated sample size was 264 children. It was increased to 657 in order to increase the accuracy of the results. The study participants were pairs of internally displaced schoolchildren and their caregivers.

### 2.3 Inclusion and exclusion criteria

Those included in the study were internally displaced schoolchildren (5–15 years) enrolled in primary schools located in the West and Littoral Regions of Cameroon and their caregivers. Internally displaced schoolchildren whose caregiver did not give their consent and those who were chronically ill were excluded from the study.

### 2.4 Data collection procedure

Data were collected using pre‐tested structured interviewer questionnaires. The caregivers of the children answered the questions on the questionnaire, which included information on the demographic and socio‐economic characteristics of the children and their caregivers, anthropometry, clinical signs of malnutrition, health‐seeking practices, morbidity, and the biochemical status of the children.

### 2.5 Sampling procedure

The West and Littoral Regions were selected for this study because these regions are among the first three that host the greatest number of internally displaced children compared to other regions in the country. Three divisions (Menoua, Bamboutos, and Mifi) in the West Region and two divisions (Wouri and Moungo) in the Littoral Region were purposely chosen because these are the most affected divisions in this region (OCHA, 2022). The names of schools with an English‐speaking subsystem of education in the respective divisions that had large numbers of displaced pupils were given by the regional delegation of basic education. A random selection of schools was made from the list. A total of 10 schools in the West Region and 12 schools in the Littoral Region were selected for the study. The multistage random sampling method was then used to ensure that the number of pupils taken from each division was proportional to the number of displaced pupils in the division. Random sampling was used in the selected schools to obtain the required sample for the study.

### 2.6 Anthropometric measurements

Weight measurement was taken using a digital electronic weighing scale calibrated in kilograms and grams (Seca model 7501017009, China). The scale was placed on a flat, hard surface. The children were requested to remove any clothing that might alter their body weight and also remove their shoes before standing on the weighing machine. It was ensured that the indicator pointer of the scale was at zero before every child was weighed. The children stood still in the middle of the scale's platform without touching anything, with the weight equally distributed on both feet. Their feet were closed, and their hands were hanging by their sides. Weight measurements were taken in duplicates, and it was ensured that the difference between the two measurements should not be more than 0.05 kg, and the average was recorded to the nearest 0.01 kg. The accuracy of the scale was checked daily using an object of standard known weight and calibrated before weighing when necessary.

Height was measured using a portable stadiometer with a movable headpiece while subjects stood erect on bare feet. The height board was always placed on a hard, flat surface against a perpendicular wall. Height was measured to the nearest 0.1 cm barefooted with the shoulders in a relaxed position and the arms hanging freely by the sides with palms facing the thigh. The subjects' heels, buttocks, and upper back were in contact with the height board. The head was held comfortably erect. The children were measured while standing by the height board with their feet flat together on the base of the board, knees straight, and heads touching the back of the board, with the headboard firmly on the head as well as on the board. Two readings were taken for each child. The headboard was then lowered to the highest point of the head with enough pressure to compress the hair. Height measurements were done in duplicate. If differences between two measurements of height for the same child exceeded 0.5 cm, measurements were repeated. The average values of the two measurements were recorded. Height measurements were taken to the nearest 0.1 cm.

MUAC was measured in millimeters using non‐stretchable tape on the left arm while the arms were hanging in a relaxed position. To avoid compression of the soft tissues, the tape was firmly but gently positioned midway between the tip of the shoulder and the tip of the elbow and recorded to the nearest 1 mm. MUAC was used to assess MUAC‐for‐age *Z*‐scores (MUACZ).

All measurements were taken according to standard procedures and recorded on the questionnaire. Height, weight, sex, and age were used to determine nutritional stats.

### 2.7 Biochemical assessment of protein, iron, and anemia

Two higher diploma laboratory technicians were recruited for the blood sample collection. About 5 mL of blood was collected from each child's veins and used to determine pre‐albumin, hemoglobin (Hb), and serum iron concentrations.

Protein status was assessed by determining serum pre‐albumin concentrations by the ELISA assay method (Vatassery et al., 1991). The reference range for pre‐albumin for children between 5–12 years (14–26 μg/dL) and 13–15 years was used (18–31 μg/dL) to indicate protein status (Chernecky & Berger, 2013). The iron status of the children was assessed by measuring their serum iron concentrations by the colorimetric method (Chernecky & Berger, 2013). The reference range for serum iron used was 50–160 μg/dL for males and 40–150 μg/dL for females (Collaborative Laboratory Services, 2013). The anemia status of the children was also assessed by measuring their Hb concentration using the Hemostat GOLD hemoglobin meter (Hemoglobin screening meter, Apex Bio, and Taiwan) by the optical reflection technique. The following cutoffs were used to define iron deficiency anemia: Hb <11.5 g/dL in children 5–11 years, and Hb <12.0 g/dL in children 12–15 years. For children 5–11 years of age, Hb concentrations between 11.0 and 11.4 g/dL were termed mild anemia. Moderate anemia was defined as the Hb concentration between 8.0 and 10.9 g/dL and severe anemia was defined as the Hb concentration lower than 8.0 g/dL. For children 12–15 years of age, mild anemia was defined when Hb levels were 11.0–11.9 g/dL, and moderate anemia was defined as 8.0–10.9 g/dL. Severe anemia was defined as Hb <8.0 g/dL (WHO, 2011). All laboratory experiments were done in the laboratory of the Bafoussam Regional Hospital, Cameroon.

### 2.8 Clinical examinations

Clinical examinations were done to diagnose the children for clinical signs of PEM, vitamin A, and iron deficiency. PEM was diagnosed based on clinical signs such as depigmentation, bilateral pitting edema, thin, dry, sparse hair, distended abdomen, and moon face. Clinical signs of vitamin A deficiency, such as the presence of Bitot's spots, xerosis, and night blindness, were assessed. Those for iron deficiency anemia that were assessed include easy fatigue, shortness of breath, dizziness, spoon‐shape nails, angular cheilitis, and pallor of the conjunctiva, tongue, or nails (Esper, 2015; Pogatshnik & Hamilton, 2011).

### 2.9 Data analysis

After collection, all data were compiled and analyzed using SPSS version 23, and appropriate statistical tests were applied. Descriptive statistics such as frequency, mean, and standard deviation were calculated and presented in tables and graphs. The WHO AnthroPlus software, which has the Growth Reference Standard for children and adolescents aged 5–19 years, was used to compute height‐for‐age *Z*‐scores (HAZ), weight‐for‐age *Z*‐scores (WAZ), body mass index‐for‐age *Z*‐scores (BMIZ), and weight‐for‐height (WHZ) *Z*‐scores based on the WHO growth reference (WHO, 2007). Severe acute malnutrition (SAM) was defined by the presence of nutritional edema on the feet or severe wasting. Moderate acute malnutrition (MAM) was defined by weight‐for‐height (WHZ) *z*‐scores >−3SD and <−2SD. Global acute malnutrition was a combination of SAM and MAM. The following cutoffs were used to interpret BMIZ: severe thinness <−3 standard deviation (SD), moderate thinness ≥−3 to <−2SD, normal ≥−2 to ≤+1 SD, overweight >+1 to ≤+2SD, obesity >+2 SD to >+3. Children whose weight‐for‐height *z*‐score, height–for–age *z*‐score, and weight‐for‐age *z*‐score were below minus 3SD below the mean based on the WHO Growth Reference. Data for 5–19 years were classified as severely wasted, severely stunted, and severely underweight, respectively. The cutoffs used to define moderate stunting, moderate underweight, and moderate wasting were ≥−3 to <−2SD (WHO, 2007). Weight‐for‐age was used to determine the nutritional status only for children between 5 and 10 years, and it was not used to determine overweight among these children (WHO, 2007). MUAC was used to calculate MUAZ and interpreted using cutoffs given by Mramba et al. (2017).

Results were expressed as means and standard deviations, frequencies, and percentages. Significant differences between the two groups were tested using *t*‐tests, McNemar's Chi‐square, and the Kruskal–Wallis test, where appropriate. The level of significance was set at *p* < .05.

### 2.10 Ethical considerations

Authorization to carry out the study was obtained from the College of Technology at the University of Bamenda. The study protocol complied with the Helsinki Declaration of 1975 and was approved by the University of Bamenda Institutional Review Board project identification number: 2021/006H/UBa/IRB. The Regional Delegate of Basic Education for the West Region gave authorization to conduct the study. Additional administrative approvals were obtained from the respective divisional delegates, inspectorates of basic education concerned, and headteachers at the selected schools. Signed informed consent was obtained from the parents or caregivers of the children. The willingness of the children to participate until the study was completed was ensured by explaining all the procedures and methods involved in the study to the children at the beginning of the study.

## 3 RESULTS

### 3.1 Demographic characteristics of the children

Table 1 shows that 51.3% of the children in the study were females, whereas the males constituted 48.7%. Most of the children (55.4%) were aged 10–15 years, while the age range of 5–9 years comprised 44.6% of the children. The mean age of the study population was 10.1 ± 1.03, with an age range between 5 and 15 years spread across classes one to six. Most of the displaced children (56%) were from the Northwest region, while 44% were from the Southwest region. The largest number of children (31.8%) were displaced in 2019. A slightly lower percentage (29.1%) were displaced in 2018. The percentage of displaced children dropped in 2020 (8.2%) and continued dropping (6.2%) in 2021.

**TABLE 1:** Demographic characteristics of the children.

Child's characteristics	Category	Frequency	Percentage
Sex of the children	Male	320	48.7
	Female	337	51.3
The age range of children	5–9 years	293	44.6
	10–15 years	364	55.4
Where the child lived before the crisis	Northwest	368	56.0
	Southwest	289	44.0
The year of child displacement	2017	162	24.7
	2018	191	29.1
	2019	209	31.8
	2020	54	8.2
	2021	41	6.2

### 3.2 Demographic characteristics and socio‐economic status of the mothers/caregivers

The mothers/caregivers of the displaced children had varying levels of education, ranging from no formal education, to university level or higher education as shown in Table 2. Regarding mothers'/caregivers' educational status, 2.1% had no formal schooling, 33.3% had primary school as their highest level of education, more than half of the mothers (55.4%) ended with a secondary education level, and only (9.1%) attended higher education. Unemployment was high among the mothers/caregivers as almost half of them were unemployed (44.7%). Nearly 48.6% of the mothers/caregivers were self‐employed, while the least proportion (7.1%) of the mothers had paid jobs. The results also showed that matrimonial status varied greatly among the mothers/caregivers of the displaced children. The greatest proportion of the mothers (69.4%) were married, followed by those who had never been married (21.9%), then widows (5.3%), and lastly the divorced mothers/caregivers with a percentage of 3.3%. The household income of the families of these displaced children was low, as most of the families (68.9%) had a monthly income below 50.000 francs, whereas the rest (23.1%) had an income ranging from 50.000 to 150.000 francs. A few families (7%) had a family income above 300.000 francs, as shown in Table 2. The households where these children lived were crowded, as 54.8% of the families had a household size between five and eight, while 32.9% of the households were overcrowded with more than eight people. Only 12.3% of the households were not crowded, as their sizes were less than five people. Most of the displaced children (60.6%) are living with their mothers. Others (37.7%) are living with a family relative. Very few of them (1.2%) were living with family friends. The table also reveals that most of the mothers (59.8%) were in the age group of 25–50 years, whereas 3% of them were below 25 years old. Only 6.7% of the mothers or caregivers were above 50 years old. The table also reveals that 26.3% of the mothers/caregivers and their children were living with another family, 70.9% were renting, and 2.7% were living in their own houses. Regarding the source of drinking water for the household, this study revealed that about 76.2% of the families had good sources of drinking water, such as tap water, spring water, and bottled water, while 18.7% drank water from bad sources, such as well and stream water. The pit toilet was the most common type of toilet used by the families of these displaced children (84.9%). A small proportion (13.2%) are using modern toilets, and a few (1.8%) do not have toilets. Most of the respondents (73.8%) were Christians.

**TABLE 2:** Demographic characteristics and socio‐economic status of the mothers/caregivers.

Parents/caregivers' characteristics	Category	Frequency	Percentage
The highest academic level of parent/caregiver	No formal education	14	2.1
	Primary	219	33.3
	Secondary	364	55.4
	Higher	60	9.1
The occupation of parent/caregiver	Formally employed	28	4.3
	Self‐employed	335	50.9
	Unemployed	294	44.7
Marital status of parent/caregiver	Married	456	69.4
	Divorced	22	3.3
	Never married	144	21.9
	Widow	35	5.3
Household income	Below 50,000	453	68.9
	50–150,000	152	23.1
	151–300,000	46	7.0
	Above 300,000	6	0.9
Number of people living in the house	Below 5	81	12.3
	5–8	360	54.8
	Above 8	216	32.9
Relationship of parent/caregiver to child	Mother	398	60.6
	Family relation	248	37.7
	Parent's friend	8	1.2
	Other	3	0.5
Age of mother/caregiver	Below 25	220	33.5
	25–50	393	59.8
	Above 50	44	70.9
Housing condition	Renting	466	70.9
	Living under another family.	173	26.3
	House owner	18	2.7
The main drinking water source	Good water source	534	81.3
	Bad water source	123	18.7
Toilet type	Pit toilet	558	84.9
	Flushing toilet	87	13.2
	No toilet	12	1.8
Religion	Christian	485	73.8
	Muslim	142	21.6
	Pagan	20	3.0
	Others	10	1.5

### 3.3 Nutritional status of schoolchildren based on *Z*‐score

#### 3.3.1 Wasting according to BMI‐for‐age *Z*‐scores of the children

**FIGURE 1:**
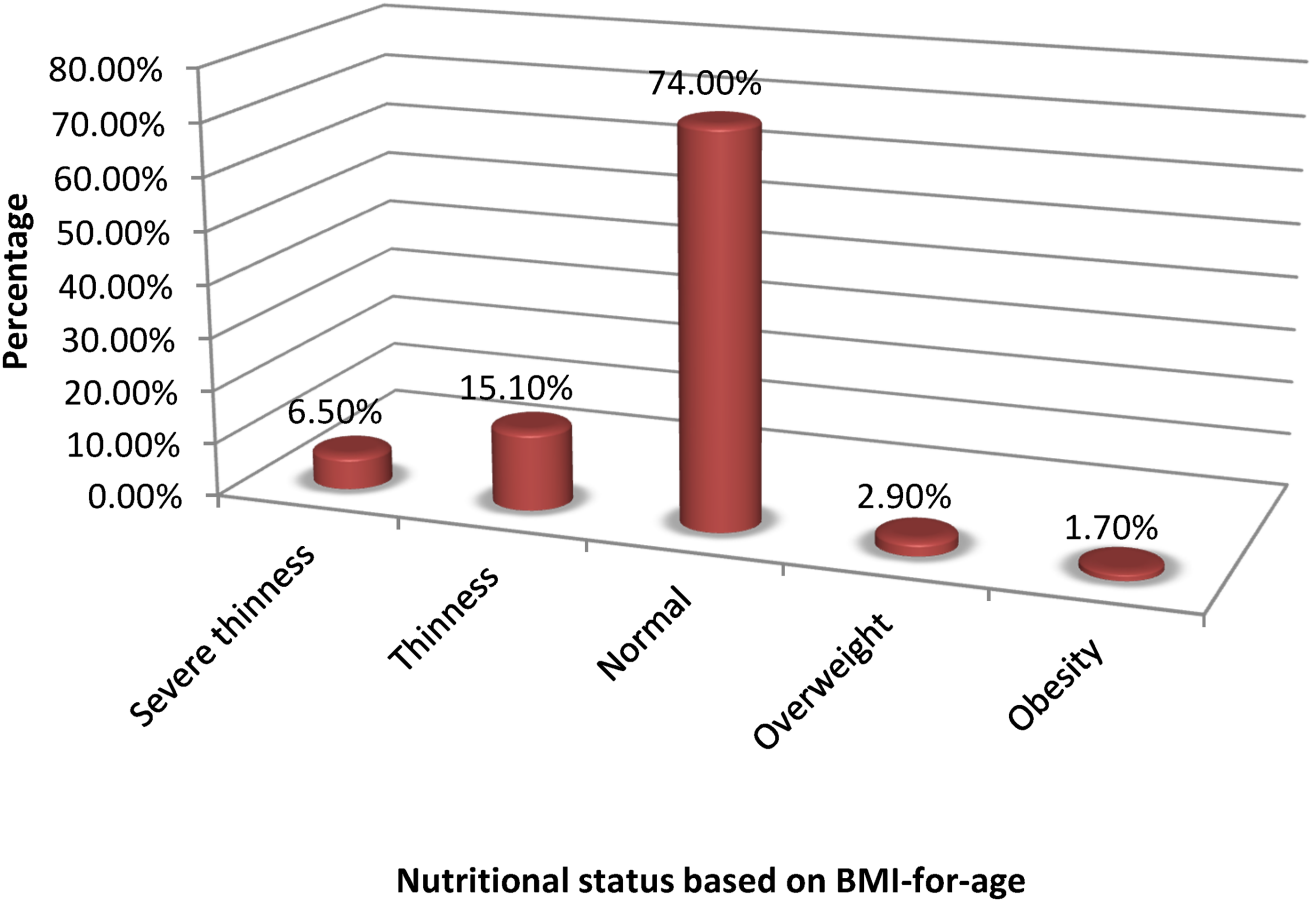
Prevalence of wasting according to BMI‐for‐age of the children.

The prevalence of severe thinness among the children was 6.5% and thinness was 15.1%, meanwhile, overweight was 2.9% and obesity was 1.7% (Figure 1). The overall prevalence of thinness was 21.6%. The minimum and maximum *Z*‐scores were −3.07 and +2.71, respectively. The mean BMIZ reported in the study was −1.45 ± 0.83. The highest rate of thinness was found in 9–10‐year‐old children, where 26.4% were thin and 1.3% were overweight. No statistically significant difference was seen between the age groups (*p* = .12). More males (24.3%) were wasted than females (18.7%). Also, the children in the West Region (24.2%) were significantly (*p* = .105) more wasted than those in the Littoral Region (19.0%).

#### 3.3.2 Wasting according to their MUAC‐for‐age *Z*‐scores

From Table 3, 7.5% of the children were severely wasted, 15.5% of them were moderately wasted, and 74.1% were normal. Wasting based on MUAC‐for‐age *Z*‐scores was the second‐highest form of malnutrition reported in the study, with an overall prevalence of 23%. More boys (25.1%) were wasted than girls (20.9%). Although more children were undernourished than overnourished, there is a coexistence of undernutrition and overnutrition among the displaced children in the West and Littoral Regions of Cameroon.

**TABLE 3:** Nutritional status of schoolchildren based on *Z*‐scores.

Underweight			Stunting			Wasting (MUAC‐for‐age *Z*‐score)		
	*N*	%		*N*	%		*N*	%
Severely underweight	40	6.1	Severely stunted	56	8.5	Severely wasted	49	7.5
Moderately underweight	92	14.0	Moderately stunted	122	18.6	Moderately wasted	102	15.5
Normal	525	79.9	Normal	479	72.9	Normal	487	74.1
						Overweight	19	2.9

#### 3.3.3 Prevalence of stunting in displaced primary schoolchildren

Stunting, which is an indicator of chronic undernutrition, was the most prevalent form of malnutrition reported in the study, with an overall prevalence of 27.1% (Table 3). Moderate stunting was observed in 18.6% and severe stunting in 8.5% of the children. About 72.9% of the children had normal height‐for‐age *Z*‐scores. The mean height‐for‐age *Z*‐score reported in the study was −1.62. The minimum and maximum *Z*‐scores were −3.21 and +2.19, respectively. Analyzing by gender and age group, the prevalence of stunting was highest in the age group of 5–6 years, where 36.6% were stunted. The lowest prevalence was noticed in the age group of 13–15 years, as only 6.2% were stunted. There was a statistically significant difference in the prevalence of stunting between age groups (*p* = .010) as it was more prevalent among the younger age group (5–9 years). Also, more boys (30.4%) were stunted than girls (23.9%). The percentage of stunted children in the West Region (28.6%) was insignificantly (*p* = .39) higher than that of children in the Littoral Region (25.6%).

#### 3.3.4 Underweight in the displaced schoolchildren

As shown in Table 3, 20.1% of the displaced schoolchildren did not attain their expected weight‐for‐age *Z*‐scores, 6.1% were severely underweight, and 14% were moderately underweight. The prevalence of underweight was highest among children who were between 7 and 8 years old (25.6%). The average weight‐for‐age *Z*‐score recorded in the study was −1.03. The least and highest *Z*‐scores were −3.04 and +2.13, respectively. Analyzing by sex shows that more boys (22.3%) were underweight than girls (18.2%). The prevalence of underweight in the West Region (21.2%) was slightly higher than the prevalence in the Littoral Region (19%).

### 3.4 Clinical signs of malnutrition

As shown in Table 4, various clinical symptoms of malnutrition were present in 9.1% of the children. Clinical signs of malnutrition were more prevalent among children in the West Region, as 10.5% of children in the West Region had at least a clinical sign of malnutrition, whereas only 7.7% of children in the Littoral Region were found to have a clinical sign of malnutrition. Thin, dry, or sparse hair was the most prevalent sign of protein deficiency, as it was noticed among 5.2% of the children; this was followed by depigmentation, which was present in 3.8% of them. Other signs of protein deficiency, such as a distended abdomen and moon face, were noticed in 0.6% of the children. Bilateral pitting edema was not observed in any of the children.

**TABLE 4:** Clinical signs of malnutrition.

Type of deficiency	Clinical sign	Frequency	Percentage
Protein deficiency	Thin, dry, or sparse hair	34	5.2
	Bilateral pitting edema	0	0
	Depigmentation	25	3.8
	Distended abdomen	4	0.6
	Moon face	4	0.6
Iron deficiency	Angular cheilitis	6	0.9
	Pallor	48	7.3
	Easy fatigue	7	1.1
	Shortness of breath	0	0.0
	Dizziness	5	0.8
Vitamin A deficiency	Night blindness	4	0.6
	Bitot's spots	10	1.5
	Xerosis	22	3.3

Signs of vitamin A deficiency such as night blindness, and xerosis were seen in 0.5%, and 3.3%, respectively. With regard to vitamin A deficiency, xerosis (3.3%) was its most prevalent sign, while Bitot's spots were not found on any of the children.

The most prevalent clinical sign of malnutrition was pallor in the skin, nail beds, palms, tongue, or inside of eyelids (7.3%), which depicts iron deficiency clinically. Other signs of iron deficiency anemia found among the children were angular cheilitis, easy fatigue, and dizziness in 0.9%, 1.1%, and 0.8%, respectively. Shortness of breath was not experienced by any of the study children.

### 3.5 Biochemical status of the children

#### 3.5.1 Prevalence of protein deficiency

**FIGURE 2:**
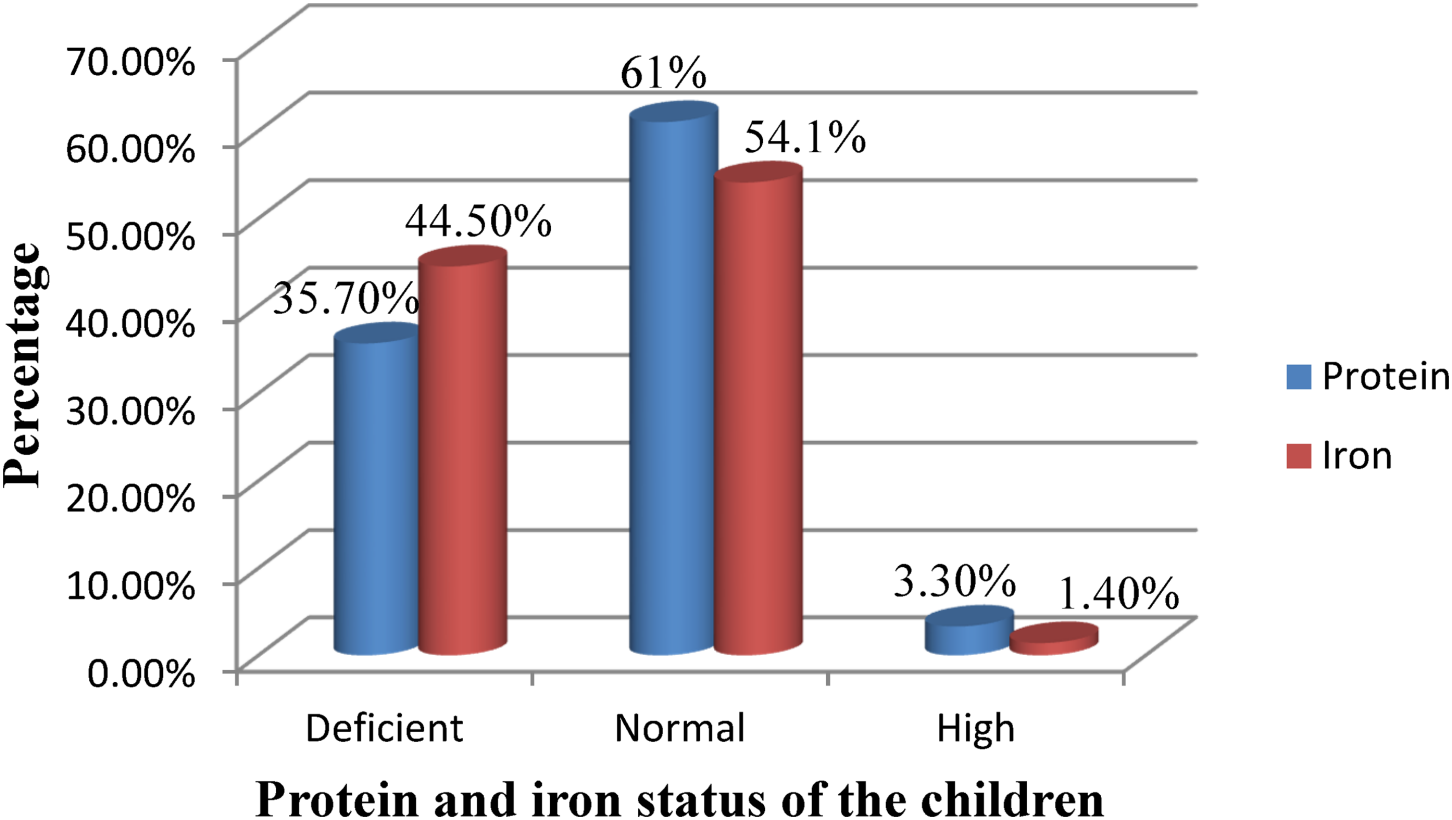
Protein and iron status of the children.

Figure 2 shows the prevalence of protein deficiency among the children based on serum pre‐albumin levels. The mean pre‐albumin level in the children was 21.7 μg/dL. Pre‐albumin levels were normal in 61% of the children, and high in 3.3%. Most (38.4%) of the protein‐deficient cases belonged to the age group of 5–9 years, and the rest (33.4%) were in the age group of 9–15. Gender‐wise, most of the protein‐deficient cases (23.9%) were males. The minimum pre‐albumin level was 11.8 μg/dL, while the maximum pre‐albumin level observed was 38.3 μg/dL. Slightly more children in the Littoral Region (36.4%) were protein deficient compared to those in the West Region (35%).

#### 3.5.2 Prevalence of iron deficiency

About 44.5% of the children were iron‐deficient (Figure 2). The mean serum iron concentration was 88.5 ± 13 μ/dL, which is within normal limits. About 54.1% of the children had normal serum iron levels, while 1.4% had unusually high levels of serum iron. Serum iron concentrations were also analyzed by gender. Iron deficiency was significantly more prevalent in females (48.4%) than in males (40.5%) (*p* = .04). The younger children (5–9 years) were more (54.3%) iron deficient than the older children 10–15 years (34.7%). The prevalence of iron deficiency was significantly (*p* = .03) higher among the children in the West Region (48.7%) than among those in the Littoral Region (40.3%).

#### 3.5.3 Prevalence of anemia

**FIGURE 3:**
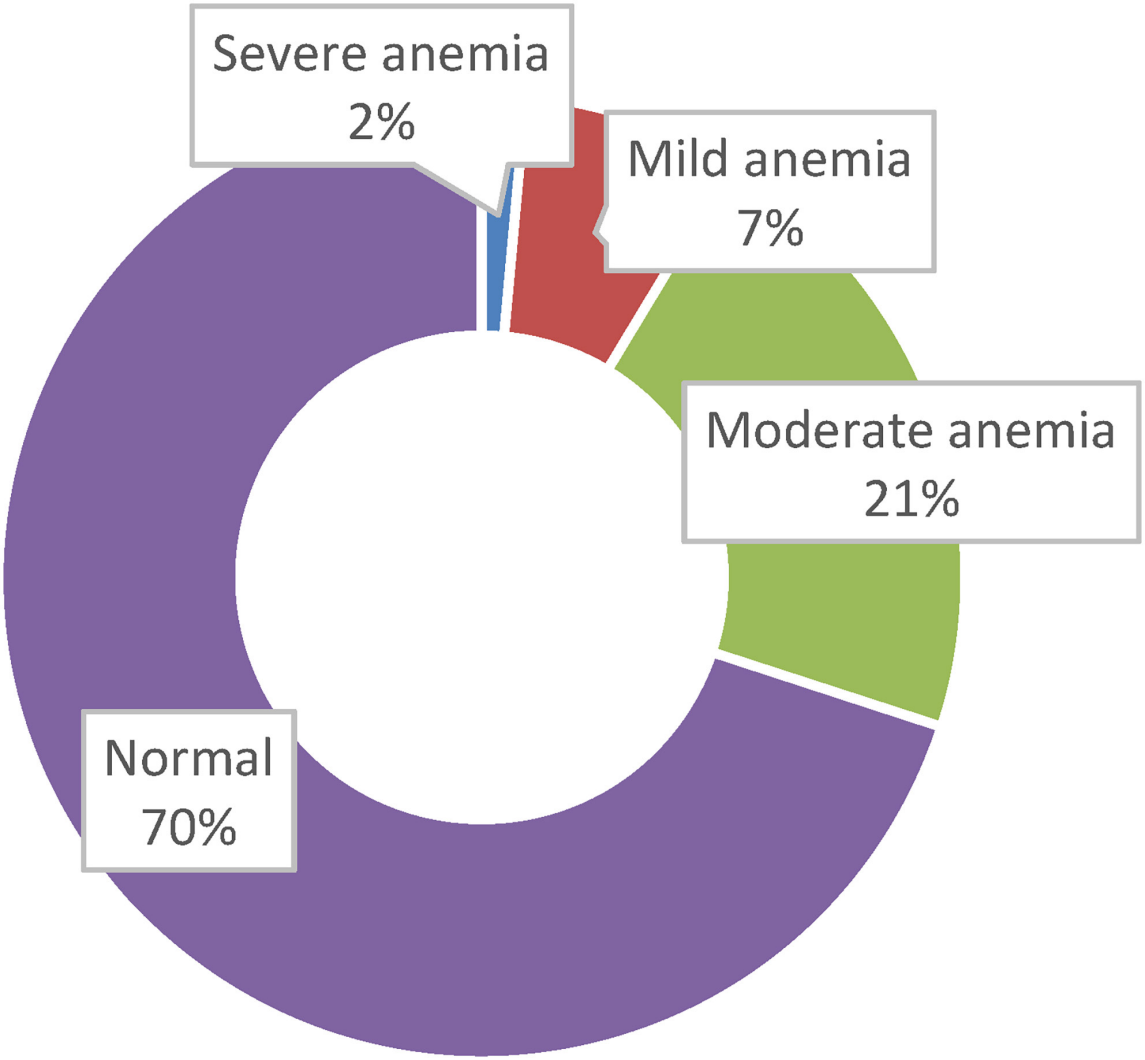
Anemia status of the children.

The prevalence of anemia among the children was 30.0%, as shown in Figure 3. The mean level of hemoglobin in the children was 12.1 g/dL. The least and highest levels of hemoglobin recorded in the study were 4.9 and 16.4 g/dL, respectively. Severe anemia had a prevalence of 2%, mild anemia was 7%, and moderate anemia was 21%. The concentrations of hemoglobin were analyzed by sex and age group. It was noticed that the prevalence of anemia among the displaced female children was higher (32.3%) than among the male children (27.7%). The prevalence of anemia among children between 5 and 9 years of age (35.5%) was higher than among those aged >9 years (24.5%). More children in the West Region (33.1%) were suffering from IDA than in the Littoral Region (26.9%).

### 3.6 Health‐seeking practices

**FIGURE 4:**
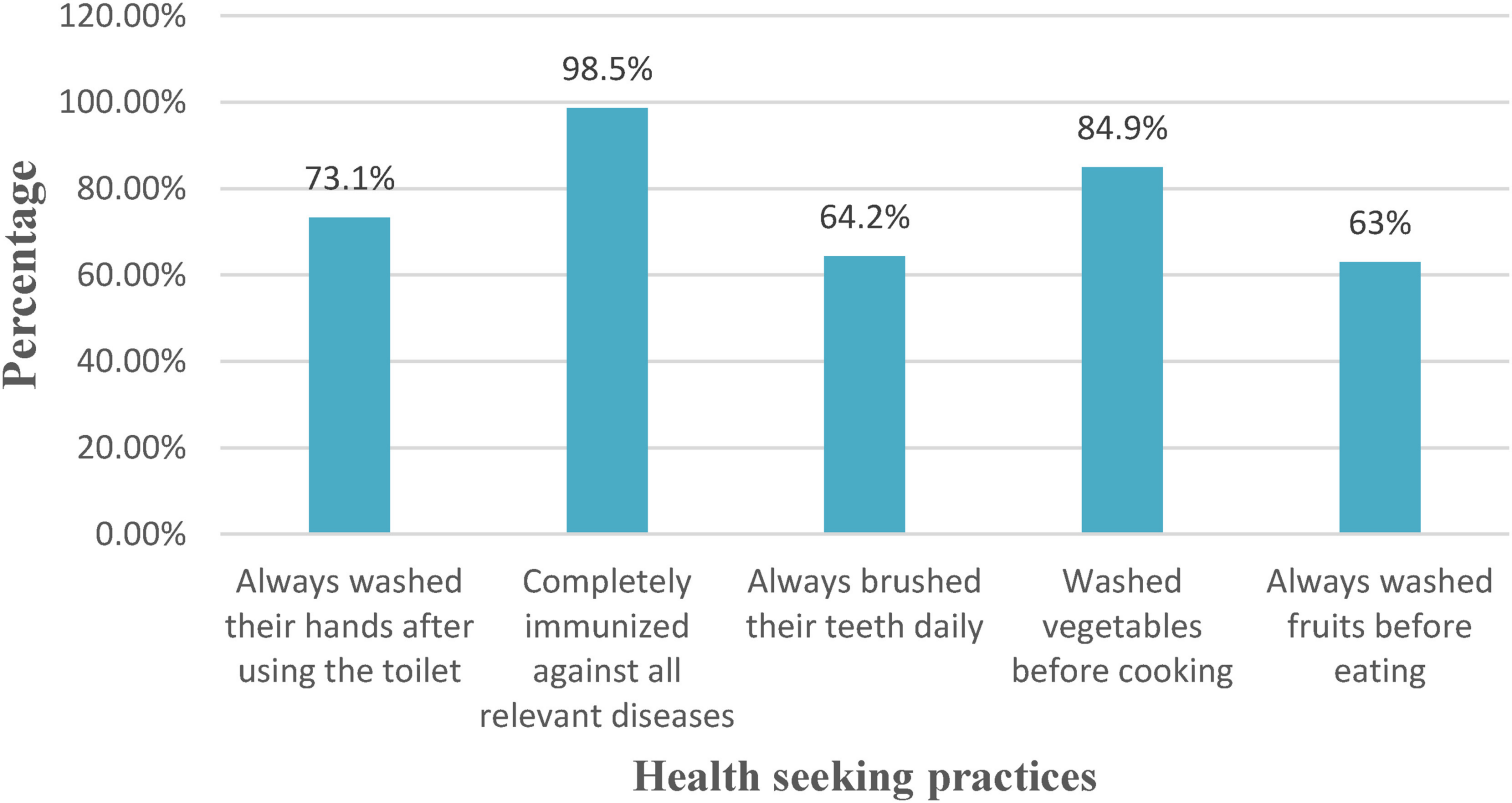
Health‐seeking practices.

From Figure 4, the hygiene status of the children revealed that about 73.1% always washed their hands after using the toilet, whereas 26.9% did not wash their hands after using the toilet. Almost all of the children 98.5% had been completely immunized against all relevant diseases. About 64.2% of subjects brushed their teeth daily, and the remaining 35.8% brushed their teeth irregularly. About 84.9% of the mothers washed vegetables before cooking, 12.1% washed vegetables after cooking, and 3% had no specific time to wash vegetables. That is, they washed vegetables either before cooking or after cooking, depending on the vegetable. Most of the pupils 63% always washed fruits before eating, while 37% of them did not wash fruits or washed fruits only sometimes before eating.

### 3.7 Health status of the children

As shown in Table 5, morbidity among the children was high because 32% of the children had suffered from a particular illness during the previous month of the study. The most common illness was respiratory tract infections (19.8%) such as common cold, cough, and pneumonia, while measles was not reported among any of the children at the time of the study. Other illnesses reported among the children included: skin infection (6.8%), stomach ache (6.4%), diarrhea (5.6%), vomiting, dental carries (5.3%), (5.2%), malaria (4.1%), and anemia (0.3%).

**TABLE 5:** Morbidity status of study children.

Disease	Frequency (*n*)	Percentage
Anemia	2	0.3
Dental Carries	35	5.3
Diarrhea	37	5.6
Vomiting	18	2.7
Measles	0	0.0
Stomach ache	42	6.4
Respiratory tract infection	130	19.8
Malaria	27	4.1
Skin infection	45	6.8

## 4 DISCUSSION

About 51.3% of the displaced children were females, whereas 48.7% were males. These results correspond to the report given by the Ministry of Public Health and WHO (2017), which documented that the number of females in Cameroon is higher than that of males. The households where these children lived were overcrowded, as many of the children (32.9%) lived in houses with a household size of more than eight people. These revelations entail that, due to the large household sizes coupled with lowincome levels reported among them, food intake was very likely to be negatively affected. The highest level of education of 33.3% of the mothers/caregivers is primary education, and 55.4%, is secondary education. These results indicate that the population may be facing difficulties in accessing relevant information, including information from sources that have been published. This may imply that the caregivers do not have adequate information on nutrition. This was confirmed by Boh et al. (2023), who noticed that the nutrition knowledge of the caregivers of the children was inadequate, and consequently, the dietary practices of their children were poor. The gaps in health‐seeking practices and nutrition knowledge noticed in this study can be used to build the capacity of the caregivers through interventions to improve the nutritional status of the children, such as nutrition education and counseling in areas including health‐seeking practices and appropriate feeding practices.

The findings of this study indicate that the displaced schoolchildren in the West and Littoral Regions of Cameroon had various nutritional challenges (undernutrition, micronutrient malnutrition, and overweight). This is in accordance with recent reports of the Demographic and Health Survey of Cameroon (Institut National de la Statistique (INS) et ICF, 2020), which revealed that about 11% of children in Cameroon are overweight and 29% are stunted. A lower rate of stunting was observed in children of the same age in the Tiko Health District, Cameroon, where only 7.5% of the children were stunted (Tabi et al., 2019). Also, a study among the children of internally displaced persons in Bamenda Health District, Cameroon, revealed that 22.1% of the children were stunted (Akeh et al., 2022). Conversely, higher rates of stunting (61.0%) were reported among schoolchildren in the Democratic Republic of Congo (Kabongo et al., 2018) and among internally displaced school‐aged children (74.5%) living in camps in the Plateau State, Nigeria (Chidiogo et al., 2022). Moreover, a systematic review that examined the nutritional status of internally displaced children in Africa revealed that 52% of them were stunted (Owoaje et al., 2016).

The most prevalent type of malnutrition determined in this study was stunting (27.1%), and this is consistent with the findings of several previous studies, which found stunting to be more prevalent than other types of malnutrition such as wasting and underweight among school‐aged children in less developed countries, including Cameroon (Best et al., 2010; Chidiogo et al., 2022; Tabi et al., 2019). These elevated rates of stunting are probably reflecting the low social and financial status of the inhabitants. The prevalence of stunting in the present study was 27.1%, which means that almost three in ten displaced school‐aged children in the West and Littoral Regions were malnourished as a consequence of chronic undernutrition. This prevalence is below the national prevalence of 31% for young children (Institut National de la Statistique (INS) et ICF, 2020), inferring that the rate of stunting in Cameroon is higher among the younger age group.

The prevalence of wasting reported in this study at 21.6% is extremely higher than what was recorded among schoolchildren in other parts of the same country. For instance, a study among primary schoolchildren in the Mount Cameroon area revealed a prevalence of wasting of 0.3% among the children (Sumbele et al., 2015). Also, a study among school‐aged children in Tiko, Cameroon, reported that only 1% of the children were wasted (Tabi et al., 2019). A systematic review of the nutritional status of children of school‐going age and adolescents in countries with low‐ and middle‐income revealed that Cameroon's school‐aged children are the least wasted when compared to other countries of the same caliber (Wrottesley et al., 2022). Therefore, the high rates of thinness observed among the children in the study may be because the children are displaced and are, hence, affected by the adverse conditions that are associated with conflicts and forcible displacement, such as food insecurity and outbreaks of contagious diseases. Also, previous studies have shown that children who are affected by conflicts are usually burdened by manifold types of malnutrition (Chidiogo et al., 2022). This prevalence of thinness (21.6%) is comparable with what was noticed among displaced children in Iraq, which was (19.2%) (Riyadh et al., 2017). A lower prevalence of thinness was reported among Senegalese school‐aged children (Fiorentino, 2015). On the other hand, extremely higher rates (58·3%) were noticed by Mulugeta et al. (2009) among Ethiopian school‐aged children and internally displaced school‐aged children (61.7%) living in the Plateau State, Nigeria (Chidiogo et al., 2022).

The prevalence of underweight (20.1%) found among the children in this study is extremely higher than the 0.7% reported by Tabi et al. (2019) among school‐aged children in the Health District of Tiko in Cameroon but lower than what was noticed among internally displaced school‐aged children (28.1%) living in the Plateau State, Nigeria (Chidiogo et al., 2022). A similar prevalence of underweight (20.4%) was reported among both internally displaced and non‐displaced children who were <5 years old in Burkina Faso (Bougma et al., 2022).

All types of malnutrition were more prevalent among the children in this study than among children of the same age in the Health District of Tiko (Tabi et al., 2019) and in the area of Mount Cameroon (Sumbele et al., 2015). The most feasible reason for the poorer results obtained in this study could be the lower socioeconomic status of the displaced children and their mothers/caregivers as the proportion of mothers/caregivers with very low household income (50.000 FCFA) was as high as 68.9%. This reaffirms the detrimental consequences of forcible displacement on the nutritional status of children.

The World Health Organization (2017) has revealed that malnutrition in children is a consequence of manifold factors, which are usually associated with the quality of food, inadequate food intake, severe and recurring communicable diseases, or a blend of these factors. These conditions, in turn, are closely associated with the general living standards and the ability of the individual to meet his/her fundamental necessities, such as sufficient food, lodging, and health care services. Hence, nutritional status assessment not only serves as a means for evaluating the health and nutritional status of children but also provides an indirect measurement of the quality of life of the whole population (WHO, 2017). The poor nutritional status of these children therefore implies that their living standards are low and their quality of life is not satisfactory.

The high prevalence of clinical signs of malnutrition among the children surveyed in the current study (9.1%) could be related to inadequate diet, diseases, inferior socio‐economic level, an unsanitary setting, poor nutrition knowledge, and a low level of education among the parents. And since these children are affected by a conflict, it further aggravates the situation, resulting in increased clinical signs of malnutrition. Though a few mothers/caregivers (7%) acknowledged that they had received humanitarian assistance in the form of food supply during the previous month of the study (Boh et al., 2023), the amount and type of aid received might be insufficient to meet the nutrient demands of the children. Hence, more humanitarian aid should be given to the households of the displaced children, especially nutritious food. Similar clinical signs and percentages were reported among schoolchildren in Sullia, Karnataka, and include pallor (12.4%), thin, dry, and sparse hair (4.2%), xerosis (2.6%), and night blindness (0.5%) (Amruth, 2012).

Clinical signs of vitamin A deficiency, such as night blindness and xerosis, were observed among the children. This prevalence of clinical signs of vitamin A deficiency noticed in this study suggests a high prevalence and a risk for vitamin A deficiency among displaced children, which is associated with illnesses such as diarrhea and measles, growth impedance, night blindness, or serious impairment of vision among children. Hence, it is necessary to determine the vitamin A status of the displaced children in the West and Littoral Regions and implement appropriate interventions to eradicate any prevailing vitamin A deficiency.

The rate of hypoalbuminemia among the displaced children, established at 35.7%, is similar to that of children living in orphanages located in the City of Douala, Cameroon. The prevalence of hypoalbuminemia among the children living in these orphanages in Douala was 34.6% (Ebongue et al., 2019). This resemblance in hypoalbuminemia prevalence is likely a result of a similarity in the living conditions and dietary intake among orphans living in orphanages and children affected by an emergency. These children are often deprived of proper dietary practices and health services, which all affect protein status negatively. A lower prevalence of protein deficiency (19.8%) was found among children less than 5 in the State of Benue, Nigeria (Abidoye & Siabofori, 2000).

The prevalence of iron deficiency reported in the current study (44.5%) is higher than what was observed by Fiorentino (2015) among children within the school‐going age and teenagers in Senegal and Cambodia (39%) and among internally displaced school‐aged children (61.7%) living in the Plateau State, Nigeria (Chidiogo et al., 2022). This reaffirms the high prevalence of micronutrient deficiencies among school‐aged children in less developed countries (Best et al., 2010). The results of this study are in concordance with those of previous studies, which have proven that iron deficiency is more prevalent among younger children because they have elevated iron requirements for growth and other metabolic activities. Among adolescents, it is more prevalent among girls who lose a significant amount of iron monthly during the process of menstruation (Development Initiatives, 2017; Khan et al., 2017; UNICEF/WHO/World Bank Group, 2018). Conversely, a cross‐sectional study conducted among school‐aged children in Libo Kemkem and Fogera districts, Amhara Regional State, Ethiopia, quantified a very low prevalence of iron deficiency (3.4%) among the children (Herrador et al., 2014).

A high prevalence of anemia (30.0%) was observed among the displaced children in the West and Littoral Regions of Cameroon. This is in concordance with the results of a cross‐sectional study conducted among school‐aged children in Ethiopia, which also stated that 30.9% of the children were anemic (Herrador et al., 2014). Again, similar trends of anemia (39%) were documented by Kana‐Sop et al. (2015) in a study carried out on young children in the West Region of Cameroon. However, the prevalence of anemia detected among children in the present study (30.0%) was greater than the prevalence reported by Sumbele et al. (2015) in seemingly healthy primary schoolchildren in the premises of Mount Cameroon (19.8%) and primary schoolchildren in the Health District of Tiko, Cameroon (5%) (Tabi et al., 2019). This is probably because these children are displaced and hence there is a loss of access to traditional foods, and a lack of food diversity further exacerbates micronutrient deficiencies in the affected population. Additionally, the availability of land and water often limits the cultivation of vegetables and fruit, which are foods that are rich in micronutrients, among displaced populations, hence limiting their access to iron‐rich foods which could have significantly improved the nutritional status of these displaced children. So, the prevalence of anemia among children can be reduced by improving the purchasing power of the mothers/caregivers of the children and ensuring that nutritious foods are always available.

In this study, 30.0% of the children had anemia, implying that anemia is a critical challenge to public health among the displaced children in these regions. Nevertheless, low levels of hemoglobin are an indicator of anemia, which is non‐specific as it is also affected by blood‐diminishing parasites, prolonged infections, and other hematological disorders (Khallafallah & Mohamed, 2012).

The results of this study highlighted an overall higher prevalence of malnutrition among younger children and adolescent girls. Therefore, the younger age group and adolescent girls should be the focal point for nutritional surveillance and interventions.

When the nutritional status of the children in this study was compared with that of non‐displaced primary schoolchildren of the same age in Cameroon and beyond, it was noticed that different forms of malnutrition were more prevalent among the children in this study. This is not surprising, as numerous previous studies confirm these results among conflict‐affected people (Bougma et al., 2022; Dago, 2021; Loewenberg, 2015).

The displaced children in this study were burdened with multiple health problems, such as malaria, respiratory tract infections, skin infections, stomach aches, diarrhea, vomiting, and dental caries. These results are similar to the findings of a systematic review done by Owoaje et al. (2016), which also noticed that the prevalence of diseases such as diarrhea, respiratory tract infections, fever, and malaria among internally displaced persons in Africa is high.

The fact that no measles was reported among the study children is an indication of the success of the measles eradication program conducted by the Cameroon government throughout the entire territory. The most prevalent illnesses noticed among the children in this current study are similar to those revealed by Amruth (2012), which include infections of the respiratory tract, skin infections, and diarrhea. About 5.3% of the children had dental caries, which can result in difficulty in mastication and reduced food intake. This finding was in contrast to a study by Amruth (2012), who reported that almost half (47.2%) of school‐aged children in Cambodia had dental caries.

Most of the children had good health‐seeking practices, as more than 60% had the appropriate practice in all cases. Almost all of the children (98.7%) had received complete immunization against all relevant diseases. This might be the reason why some preventable infectious diseases, such as measles, were not reported among the displaced children. In the current study, most of the children and their caregivers had good health‐seeking practices, unlike most of the mothers of under‐five children in the Calabar South local area of the government, Nigeria, who had inappropriate health‐seeking behavior toward their children (Jemide et al., 2016). Those who did not have good practices were exposed to diseases such as diarrhea and dental caries, and this may account for the high prevalence of these diseases among the children. The unsanitary practices noticed among the children and their caregivers might be due to ignorance or a lack of the necessary facilities, such as portable tap water to wash their hands, fruits, or vegetables at the right time. On a long‐term basis, ensuring constant availability of potable water and hygienic modalities is among the fundamental interventions to promote health among displaced children. Hygiene and sanitation are well‐recognized keys to the good health and nutritional status of children (United Nations, 2016). Also, executing regular nutritional monitoring of displaced schoolchildren as part of a school health program can be instrumental in bringing about a much‐required improvement in the nutritional status of the children.

## 5 CONCLUSION

The study children constituted more girls (51.3%) than boys (48.7%). The years 2018 and 2019 were the peaks of displacement (29.1% and 31.8%, respectively). Various clinical symptoms of malnutrition were present among 9.1% of the children. The most prevalent clinical sign of malnutrition was pallor (7.3%), which signifies iron deficiency, while bilateral pitting edema and Bitot's spots were not noticed among any of the children. The proportion of children who were wasted, underweight, and stunted was 23%, 20.1%, and 27.1%, respectively. Morbidity among the children was high since 32% of the children had suffered from a particular illness during the past month. The most common illness (19.8%) was respiratory tract infections such as common colds, coughs, and pneumonia, while measles was not reported among any of the children at the time of the study. Immunization (98.5%) was the most common health‐seeking practice, meanwhile washing fruits before eating was the most neglected (63%) health‐seeking practice among the children. About 44.4% of them had low serum iron content, and the prevalence of anemia was 30.0%. About 35.7% of them had poor protein status. The prevailing scenario of the health and nutritional status of the displaced children in the West and Littoral Regions is very unsatisfactory and might be a reflection of the low nutrient intake of the children. Interventions to improve nutritional status should be intensified specifically among displaced families and their hosts as a strategy for preventing malnutrition in the short term and diet‐related diseases in the long term among the children. Supplementary feeding of the children with food that is particularly rich in protein, iron, and vitamin A is required.

## AUTHOR CONTRIBUTIONS

**Boh Mirabelle Nwachan:** Conceptualization (equal); data curation (equal); formal analysis (lead); funding acquisition (lead); methodology (equal); software (equal); writing – original draft (lead); writing – review and editing (equal). **Aba Richard Ejoh:** Conceptualization (equal); data curation (equal); formal analysis (supporting); investigation (supporting); methodology (supporting); project administration (lead); resources (supporting); supervision (lead); writing – original draft (supporting); writing – review and editing (equal). **Ngangmou Theirry Noumo:** Formal analysis (equal); methodology (equal); writing – review and editing (equal).

## FUNDING INFORMATION

No funding was provided to carry out this study. All expenditures were solely borne by Mrs. Nwachan B.M.

## CONFLICT OF INTEREST STATEMENT

The authors declare that they have no conflict of interest.

## Data Availability

The data that support the findings of this study are available on request from the corresponding author (ejohrab62@gmail.com).

## References

[fsn34068-bib-0044] Abidoye, R. O. , & Bofori, S. (2000). A study of prevalence of protein energy malnutrition among 0–5 years in rural Benue State, Nigeria. Nutrition and Health, 13(4), 235–347. 10768411

[fsn34068-bib-0001] Akeh, M. L. , Tendongfor, N. , Nchung, A. J. , Chipili, G. , Mbhenyane, X. , & Tambe, A. B. (2022). Magnitude and predictors of malnutrition among internally displaced persons' children 6–59 months in Bamenda Health District of Cameroon: A community‐based cross‐sectional study. Journal of Nutrition and Health. 10.1177/02601060221132134 36237133

[fsn34068-bib-0002] Amruth, M. (2012). A study on the nutritional status and risk factors for malnutrition among primary school children in Sullia, Karnataka (Master's thesis). Shridevi Institute of Medical Sciences & Research Hospital, Department of Community Medicine.

[fsn34068-bib-0047] Benneh, G. , & DeLancey, M. W. (2004). Cameroon: Culture, history and people . https://www.britanica.com

[fsn34068-bib-0003] Best, C. , Neufingerl, N. , van Geel, L. , van den Briel, T. , & Osendarp, S. (2010). The nutritional status of school‐aged children: Why should we care? Food and Nutrition Bulletin, 31(3), 400–417. 10.1177/156482651003100303 20973461

[fsn34068-bib-0042] Boh, N. M. , Aba, E. R. , & Lemfor, C. B. (2023). Dietary practices and nutrient intake of internally displaced school children in the West Region of Cameroon. International Journal of Food Science, 2023, 9954118. 10.1155/2023/9954118 36852392 PMC9966561

[fsn34068-bib-0004] Bougma, S. , Hama‐Ba, F. , Garanet, F. , & Savadogo, A. (2022). Nutritional status of children under five years of age among internally displaced populations and non‐displaced in Burkina Faso. Journal of Food and Nutrition Research, 10(7), 449–458. 10.12691/jfnr-10-7-2

[fsn34068-bib-0005] Chernecky, C. C. , & Berger, B. J. (2013). Laboratory tests and diagnostic procedures (6th ed.). Elsevier/Sauders.

[fsn34068-bib-0006] Chidiogo, L. U. , Franca, O. O. , Uju, I. , & Nnubia, E. J. N. (2022). Assessment of the nutritional status of school‐aged children in internally displaced persons camps in Plateau State, Nigeria. Nigerian Journal of Nutritional Sciences, 43(1), 160–170.

[fsn34068-bib-0037] Collaborative Laboratory Services . (2013). Laboratory procedure manual. Albumin in refrigerated serum . NHANES 2013–2014.

[fsn34068-bib-0007] Dago, E. (2021). Armed conflicts and food insecurity‐a short literature review (pp. 1–14). INRAe/CIRAD/Alliance of Biodiversity International and CIAT. https://hdl.handle.net/10568/114586

[fsn34068-bib-0008] Development Initiatives . (2017). Global nutrition report 2017: Nourishing the SDGs. Development Initiatives. http://globalnutritionreport.org

[fsn34068-bib-0048] Ebongue, C. O. , Koum, D. K. , Penda, C. I. , Nda‐Mefoo, J. P. , WAnye, F. , Eloumou, S. A. , et al. (2019). Assessment of the nutritional status of children living in Orphanages in the City of Douala, Cameroon. International Journal of child health and nutrition, 8(1), 1–9. 10.6000/1929-4247.2019.08.01.1

[fsn34068-bib-0039] Esper, D. H. (2015). Utilization of nutrition‐focused physical assessment in identifying micronutrient deficiencies. Journal of Nutrition in Clinical Practice, 30(2), 194–202. 10.1177/0884533615573054 25829342

[fsn34068-bib-0009] Fiorentino, M. (2015). Malnutrition in school‐aged children and adolescents in Senegal and Cambodia: Public health issues and interventions. Food and Nutrition (PhD thesis). Université Montpellier, English, NNT.

[fsn34068-bib-0010] Fisher, A. A. , Laing, J. E. , Stoeckel, J. E. , & Townsend, J. W. (1991). Handbook for family planning operations research designs (2nd ed., p. 22). The Population Council, Guilford Press. 10.31899/RH10.1039

[fsn34068-bib-0011] FSIN . (2021). Global report on food crises. Joint analysis for better decisions. FSIN. http://www.wfp.org

[fsn34068-bib-0012] Global Nutrition Report . (2018). Nutrition country profile Cameroon. Global Nutrition Report. globalnutritionreport.org

[fsn34068-bib-0013] Herrador, Z. , Sordo, L. , Gadisa, E. , Buño, A. , Gómez‐Rioja, R. , Iturzaeta, J. M. , de Armas, L. F. , Benito, A. , Aseffa, A. , Moreno, J. , Cañavate, C. , & Custodio, E. (2014). Micronutrient deficiencies and related factors in school‐aged children in Ethiopia: A cross‐sectional study in Libo Kemkem and Fogera districts, Amhara Regional State. PLoS One, 9(12), e112858. 10.1371/journal.pone.0112858 25546056 PMC4278675

[fsn34068-bib-0049] Institut National de la Statistique (INS) et ICF . (2020). Enquête demographique et de sante du Cameroun 2018 . Cameroun et Rockville, INS et ICF, Yaounde, Cameroun.

[fsn34068-bib-0046] Jemide, J. O. , Edet, E. E. , Udoh, E. E. , & Ene‐Obong, H. N. (2016). Health‐seeking behaviours of mothers of under‐five children in Calabar South Local Government area, Cross River State, Nigeria. International Journal of Home Sciences and Research, 6(5), 214–222.

[fsn34068-bib-0014] Kabongo, M. M. , Linsuke, S. , Baloji, S. , Mukunda, F. , Raqual, I. L. , Stauber, C. , Van Geetrude, J. P. , & Lutumba, P. (2018). Schistosoma Manson infection and its association with nutrition and health outcomes: A household survey in school‐aged children living in Kasansa, Democratic Republic of the Congo. Pan African Medical Journal, 31(197), 1937–8688. 10.11604/pamj.2018.31.197.16.364 PMC6488962 31086641

[fsn34068-bib-0043] Kana‐sop, M. M. , Mananga, M. J. , Tetanye, E. , & Gouado, I. (2015). Risk factors of anaemia among young children in rural Cameroon. International Journal of Current Microbiology and Applied Sciences, 4(3), 925–935.

[fsn34068-bib-0015] Kathryn, G. , & Begum, D. (2011). Long‐term consequences of stunting in early life. Journal of Maternal and Child Nutrition, 7(3), 5–18. 10.1111/j.1740-8709.2011.00349.x 21929633 PMC6860846

[fsn34068-bib-0045] Khallafallah, A. A. , & Mohamed, M. (2012). Nutritional Anemia. IntechOpen. 10.5772/30613

[fsn34068-bib-0016] Khan, A. , Khan, S. , Zia‐ul‐Islam, S. , Tauqeer, A. M. , Riffat , & Khan, M. (2017). Causes, signs, and symptoms of malnutrition among the children. Journal of Nutrition and Human Health, 1(1), 24–27. 10.35841/nutrition-human-health.1.1.24-27

[fsn34068-bib-0017] Loewenberg, S. R. (2015). Conflicts worsen global hunger crisis worldwide, aid agencies are dealing with larger, more complex food insecurity crises than ever before, exacerbated by conflicts and severe weather events. Sam Loewenberg Reports, 386(10005), 1719–1721. 10.1016/S0140-6736(15)00734-5 26545421

[fsn34068-bib-0018] Ministry of Public Health & WHO . (2017). Health population denominators 2017. Ministry of Public Health & WHO. https://dhsprogram.com

[fsn34068-bib-0041] Mramba, L. , Ngari, M. , Mwangome, M. , Muchai, L. , Bauni, E. , Walker, A. S. , Gibb, D. M. , Fegan, G. , & Berkley, J. A. (2017). A growth reference for mid upper arm circumference for age among school‐age children and adolescents, and validation for mortality, growth curve construction, and longitudinal cohort study. Biomedical Journal of Clinical Research, 358, j3423. 10.1136/bmj.j3423 PMC5541507 28774873

[fsn34068-bib-0019] Mulugeta, A. , Hagos, F. , Stoecker, B. , Kruseman, G. , Linderhof, V. , Abraha, Z. , Yohannes, M. , & Samuel, G. G. (2009). Nutritional status of adolescent girls from rural communities of Tigray, northern Ethiopia. The Ethiopian Journal of Health Development, 23, 5–11. 10.4314/ejhd.v23i1.44831

[fsn34068-bib-0020] OCHA . (2019). Cameroon: North‐West and South‐West Situation Report No. 12. As of 31 October 2019 . www.unocha.org.www.reliefweb.int

[fsn34068-bib-0035] OCHA . (2022). Cameroon: North‐West and South‐West Situation Report No. 45. As of July 2023 . www.unocha.org.www.reliefweb.int. Annual Report 2022|OCHA (unocha.org)

[fsn34068-bib-0021] Owoaje, E. T. , Uchendu, O. C. , Ajayi, T. O. , & Cadmus, E. O. (2016). A review of the health problems of the internally displaced persons in Africa. The Nigerian Postgraduate Medical Journal, 23, 161–171. 10.4103/1117-1936.196242 28000636

[fsn34068-bib-0040] Pogatshnik, C. , & Hamilton, C. (2011). Nutrition‐focused physical examination: Skin nails hair eyes and oral cavity, Support Line, 33(2), 7–13.

[fsn34068-bib-0036] Prinzo, Z. W. , & de Benoist, B. (2002). Meeting the challenges of micronutrient deficiencies in emergency‐affected populations. Proceedings of the Nutrition Society, 61(2), 251–257. 10.1079/PNS2002151 12133207

[fsn34068-bib-0022] Riyadh, L. , Hala, A. l. S. , Saba, D. , & Qudama, A. (2017). Nutritional status assessment of internally displaced children in “dream City”‐ Iraq. Journal of Food and Nutrition Sciences, 5(3), 122–130. 10.11648/j.jfns.20170503.19

[fsn34068-bib-0023] Salami, B. , Iwuagwu, S. , Amodu, O. , Tulli, M. , Ndikom, C. , Gommaa, H. , Lavin, T. , & Kariwo, M. (2020). The health of internally displaced children in sub‐Saharan Africa: A scoping review. Biomedical Journal of Global Health, 5, e002584. 10.1136/bmjgh-2020-002584 PMC7454178 32859650

[fsn34068-bib-0024] Sumbele, I. U. , Kimbi, H. K. , Ndamukong‐Nyanga, J. L. , Nweboh, M. , Anchang‐Kimbi, J. K. , Lum, E. , Nana, Y. , Ndamukong, K. K. J. , & Lehman, L. G. (2015). Malarial anemia and anemia severity in apparently healthy primary school children in urban and rural settings in the Mount Cameroon area: Cross‐sectional survey. PLoS One, 10(4), e0123459. 10.1371/journal.pone.0123549 25893500 PMC4403990

[fsn34068-bib-0025] Tabi, E. S. B. , Cumber, S. N. , Juma, K. O. , Ngoh, E. A. , Achidi, E. A. , & Eyong, E. M. (2019). A cross‐sectional survey on the prevalence of anemia and malnutrition in primary school children in the Tiko Health District, Cameroon. The Pan African Medical Journal, 32(111), 5–7. 10.11604/pamj.2019.32.111.15728 PMC6560948 31223401

[fsn34068-bib-0026] Teo, C. H. , Chin, Y. S. , Lim, P. Y. , Masrom, S. A. H. , & Shariff, Z. M. (2021). Impacts of a school‐based intervention that incorporates nutrition education and a supportive healthy school canteen environment among primary school children in Malaysia. Nutrients, 13(5), 1712. 10.3390/nu13051712 34070053 PMC8158127

[fsn34068-bib-0027] UNICEF/WHO/World Bank Group . (2018). Joint child malnutrition estimates. Levels and trends in child malnutrition. Key findings of the 2018 edition of the joint child malnutrition estimates . https://data.unicef.org

[fsn34068-bib-0028] United Nations . (2016). End Hunger: Sustained Development Goal (SDG) 2 . https://sustainabledevelopment.un.org/focussdgs.html

[fsn34068-bib-0029] Vatassery, G. T. , Quach, H. T. , Smith, W. E. , & Eckfeldt, J. H. (1991). A sensitive assay of transthyretin (pre‐albumin) in human cerebrospinal fluid in nanogram amounts by ELISA. Clinical Chim Acta, 1991(197), 19–25. 10.1016/0009-8981(91)90344-c 2044212

[fsn34068-bib-0030] Vos, R. , Jackson, J. , James, S. , & Sánchez, M. V. (2020). Refugees and conflict‐affected people integrating displaced communities into food systems. In 2020 Global food policy report: Building inclusive food systems (pp. 46–53). International Food Policy Research Institute (IFPRI). 10.2499/9780896293670

[fsn34068-bib-0031] WHO . (2007). WHO child growth standards and the identification of severe acute malnutrition in infants and children. A Joint Statement by the World Health Organization and the United Nations Children's Fund. WHO. www.whoo.int 24809116

[fsn34068-bib-0032] WHO . (2017). Nutrition in the WHO African region. WHO. www.who.int

[fsn34068-bib-0038] WHO . (2011). Hemoglobin concentrations for the diagnosis of anemia and assessment of severity Vitamin and Mineral Nutrition Information System Geneva World Health Organization 2011 . Geneva (WHO/NMMH/NHM/MNM/111).

[fsn34068-bib-0033] Wrottesley, S. V. , Mates, E. , Brennan, E. , Bijalwan, V. , Menezes, R. , Ray, S. , Ali, Z. , Amirhossein Yarparvar, A. , Sharma, D. , & Lelijveld, N. (2022). Nutritional status of school‐age children and adolescents in low‐ and middle‐income countries across seven global regions: A synthesis of scoping reviews. Journal of Public Health Nutrition, 26, 63–95. 10.1017/S1368980022000350 35156607 PMC11077463

[fsn34068-bib-0034] Zerga, A. A. , Tadesse, S. E. , Ayele, F. Y. , & Ayele, S. Z. (2022). Impact of malnutrition on the academic performance of school children in Ethiopia: A systematic review and meta‐analysis. SAGE Open Medicine, 2022(10), 205031212211223. 10.1177/20503121221122398 PMC9500247 36161209

